# Supplementary figures and images for: Interdependence of plasma membrane nanoscale dynamics of a kinase and its cognate substrate underlies Arabidopsis response to viral infection
Source: eLife. 2025 May 2;12:RP90309. doi: 10.7554/eLife.90309 (PMC12048157; doi:10.7554/eLife.90309)

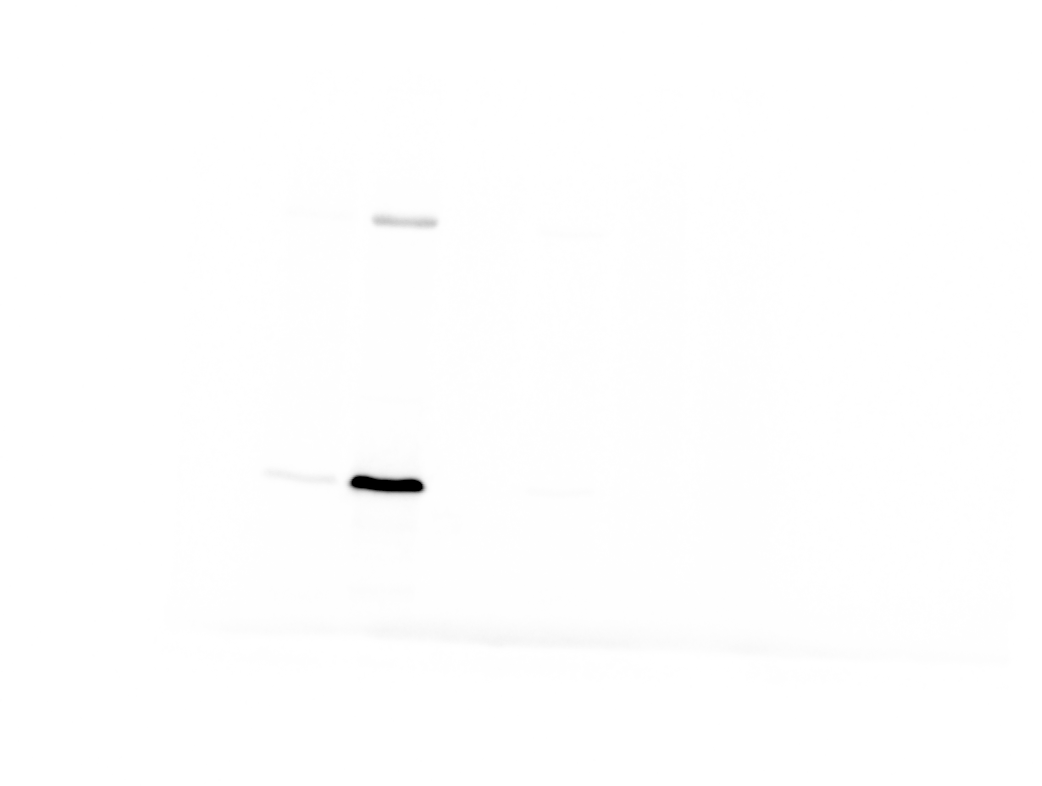

Supplement: Figure 1—source data 1. [file elife-90309-fig1-data1.zip › Figure 1 source data 1/Fig1F_blot.tif]

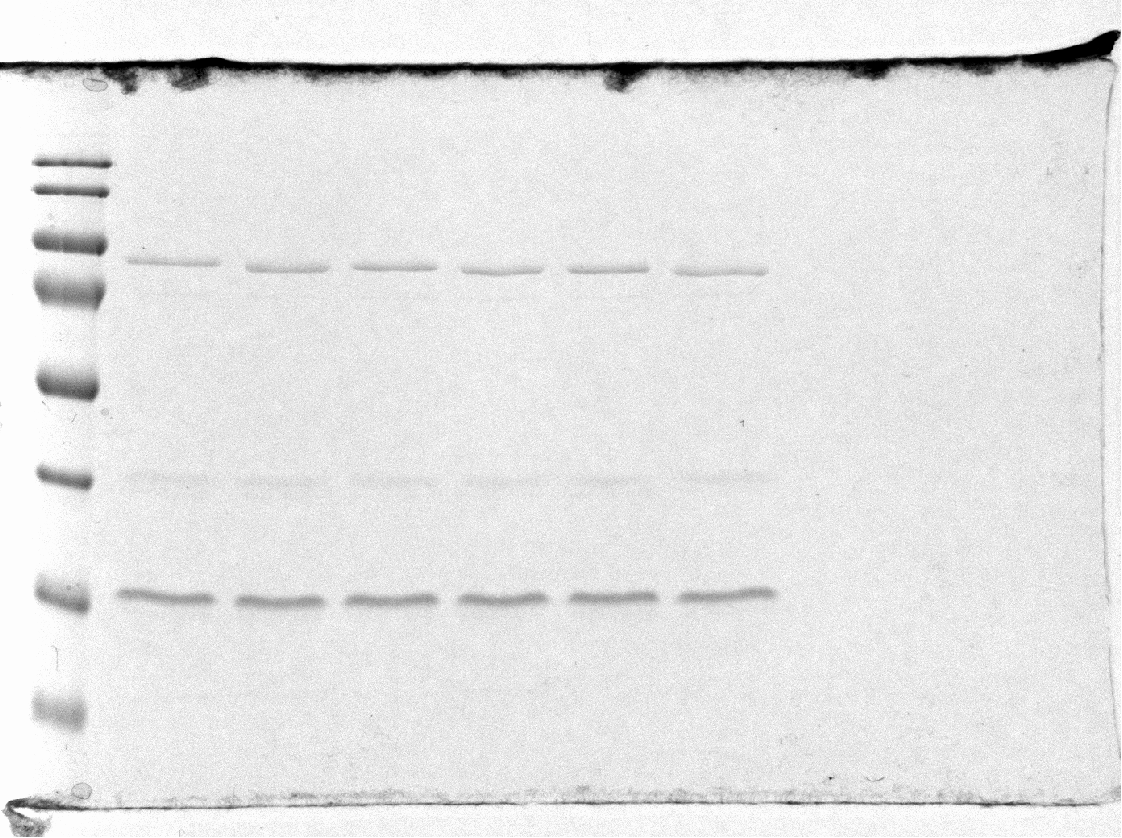

Supplement: Figure 1—source data 1. [file elife-90309-fig1-data1.zip › Figure 1 source data 1/Fig1F_coomassie.tif]

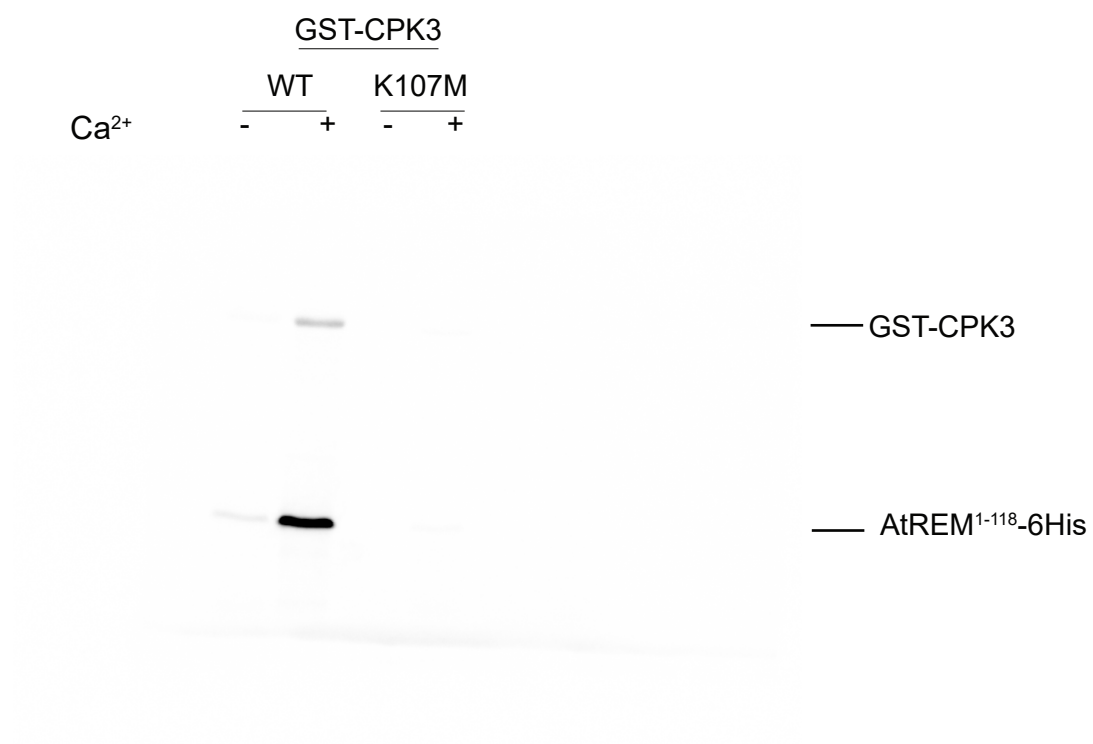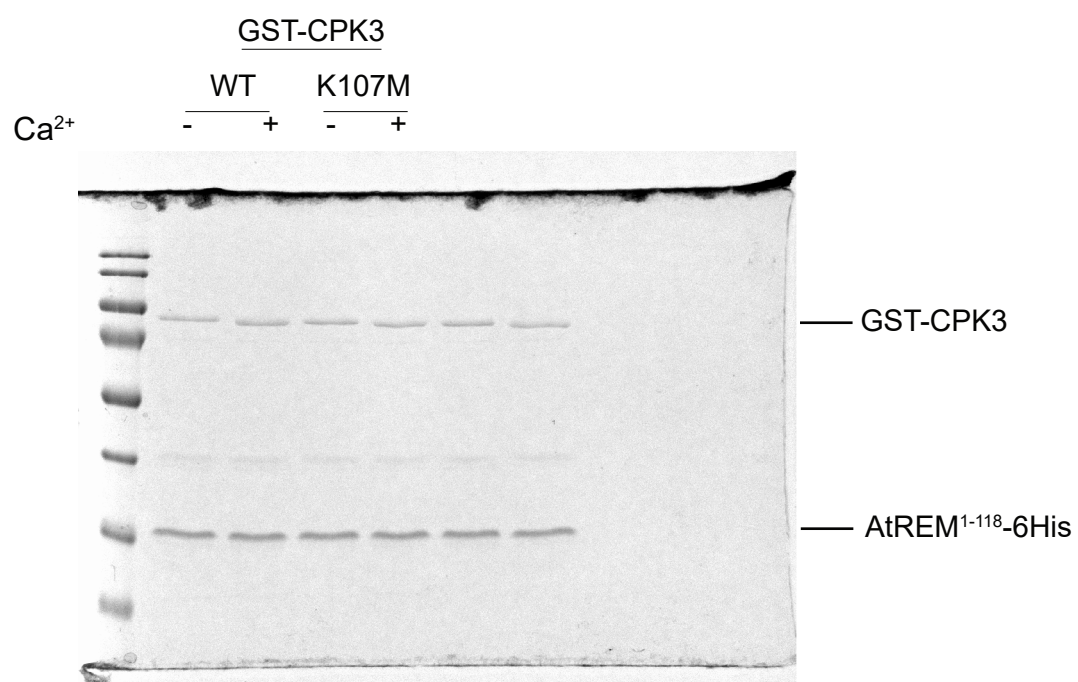

Supplement: Figure 1—source data 2. [file elife-90309-fig1-data2.zip › Figure 1 source data 2/Figure 1 source data 2.pdf]

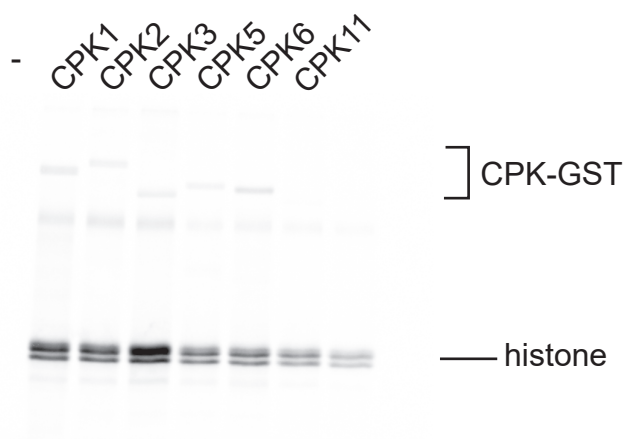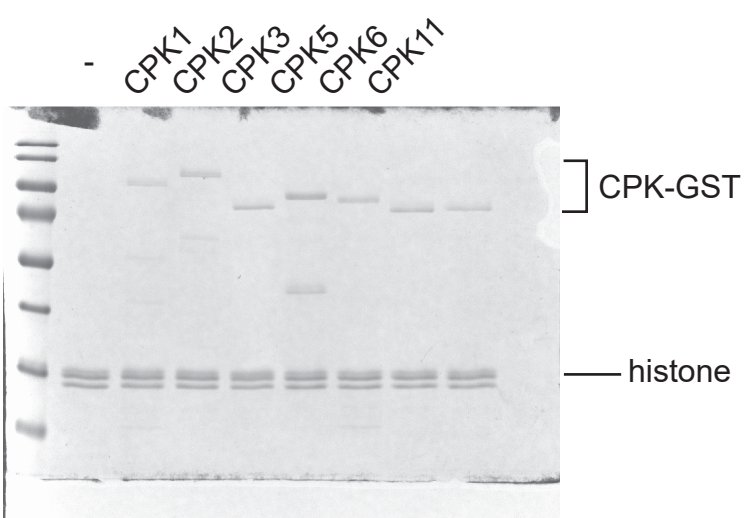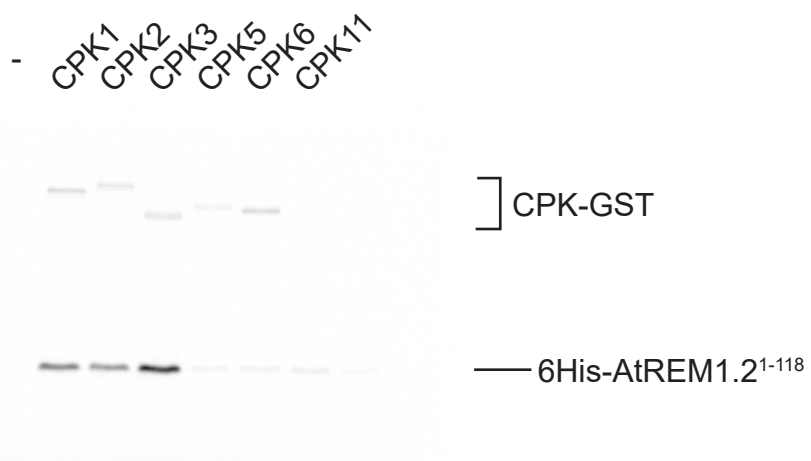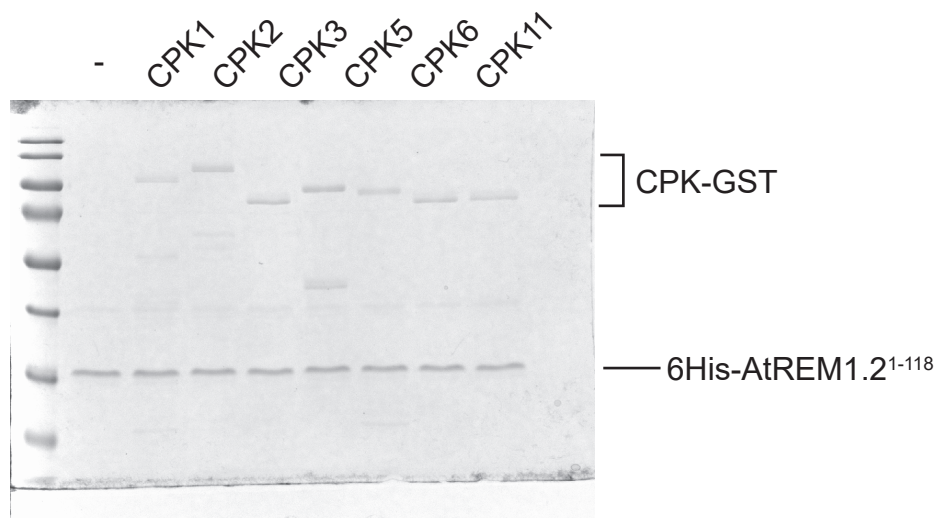

Supplement: Figure 1—figure supplement 2—source data 2. [file elife-90309-fig1-figsupp2-data2.zip › Figure 1 source data 4/Figure 1 source data 4.pdf]

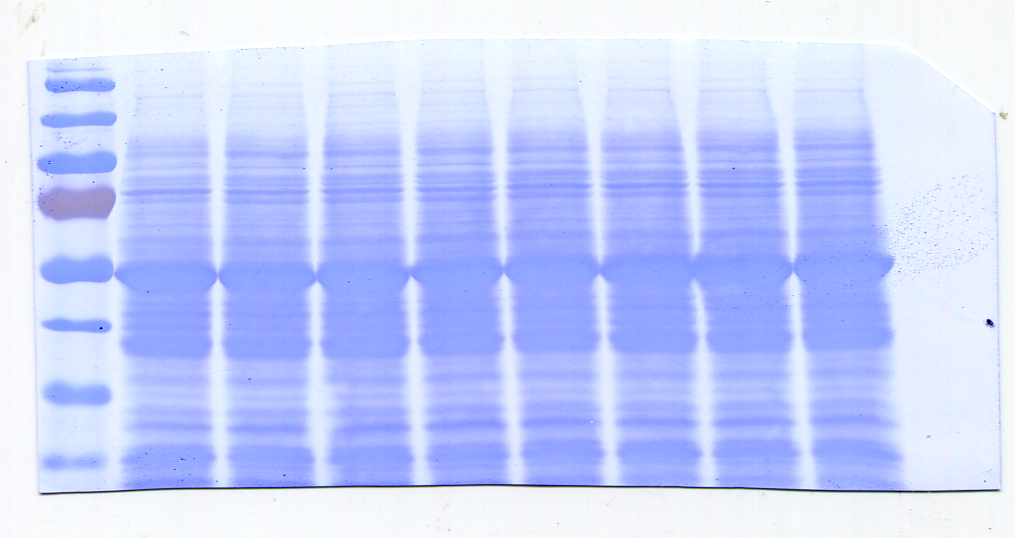

Supplement: Figure 1—figure supplement 3—source data 1. [file elife-90309-fig1-figsupp3-data1.zip › Figure 1 source data 5/Fig1sup3_CPK3_coom.tif]

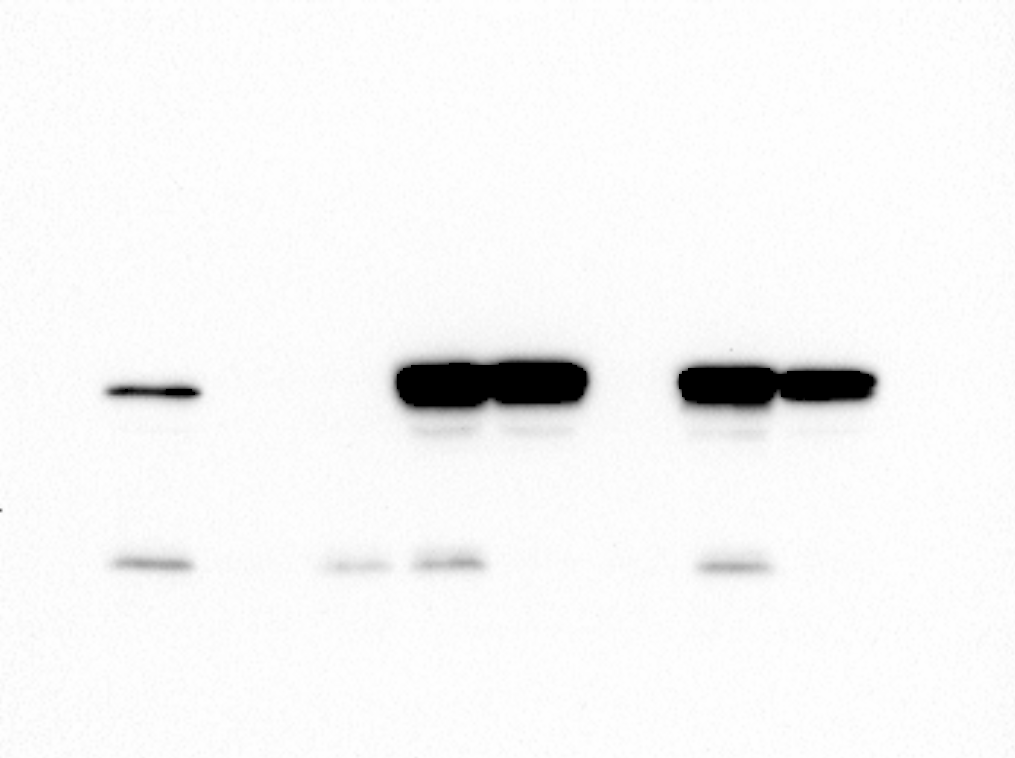

Supplement: Figure 1—figure supplement 3—source data 1. [file elife-90309-fig1-figsupp3-data1.zip › Figure 1 source data 5/Fig1sup3_CPK3_WB.tif]

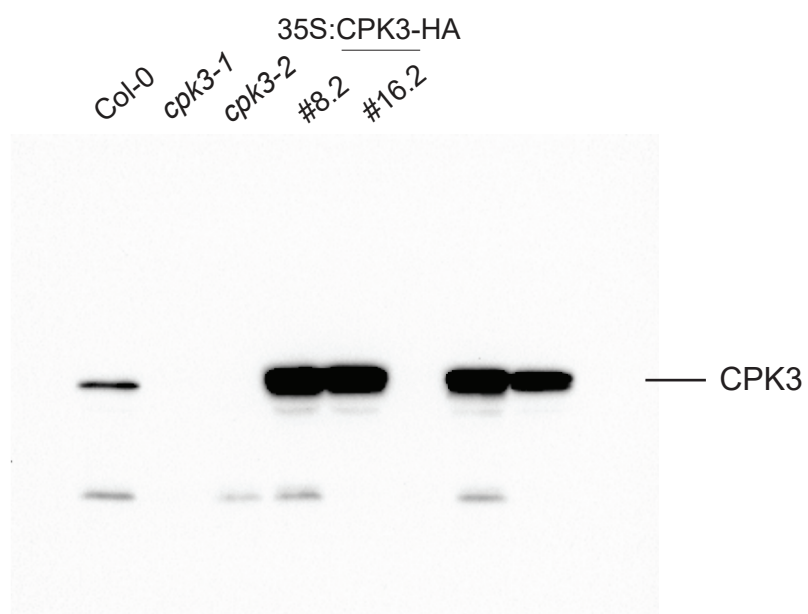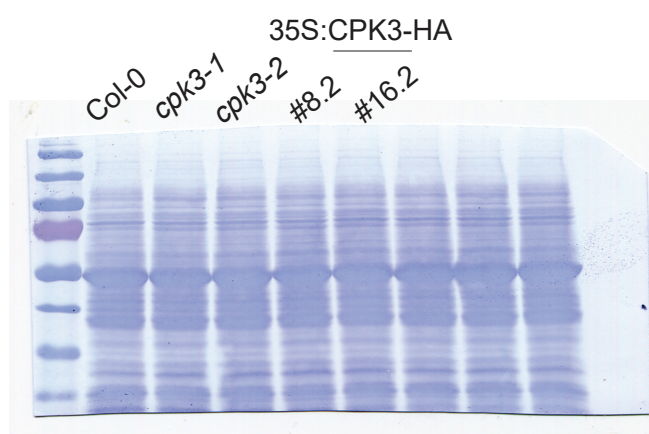

Supplement: Figure 1—figure supplement 3—source data 2. [file elife-90309-fig1-figsupp3-data2.zip › Figure 1 source data 6/Figure 1 source data 6.pdf]

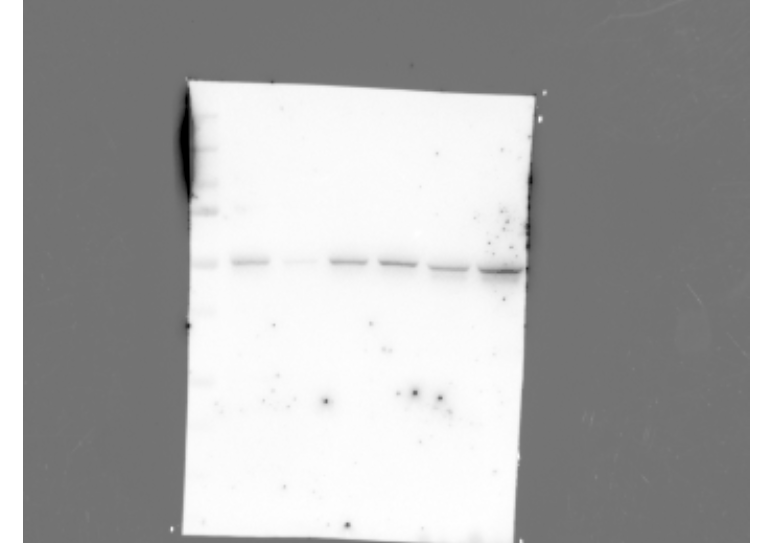

Supplement: Figure 2—figure supplement 1—source data 2. [file elife-90309-fig2-figsupp1-data2.zip › Figure 2 source data 1/membrane_antiCPK3.tif]

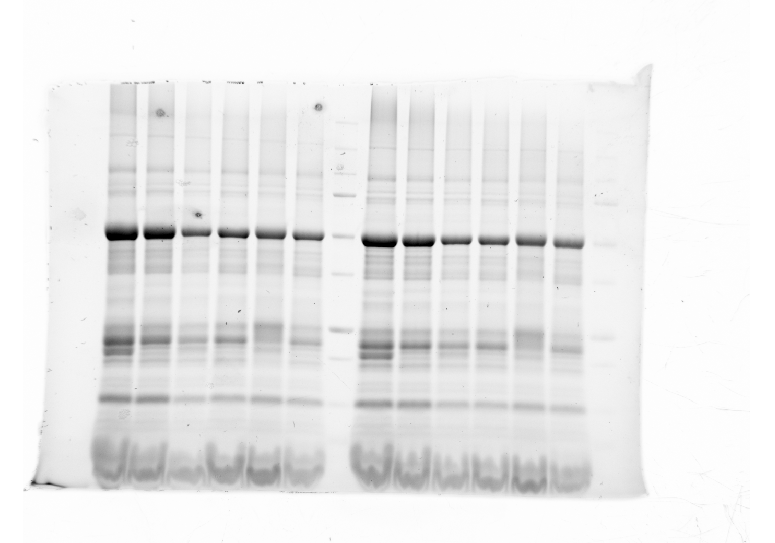

Supplement: Figure 2—figure supplement 1—source data 2. [file elife-90309-fig2-figsupp1-data2.zip › Figure 2 source data 1/stain_free_gel.tif]

|            |    |   |    |   |    |   |
|------------|----|---|----|---|----|---|
| Replicates | #1 |   | #2 |   | #3 |   |
| PIAMV      | -  | + | -  | + | -  | + |

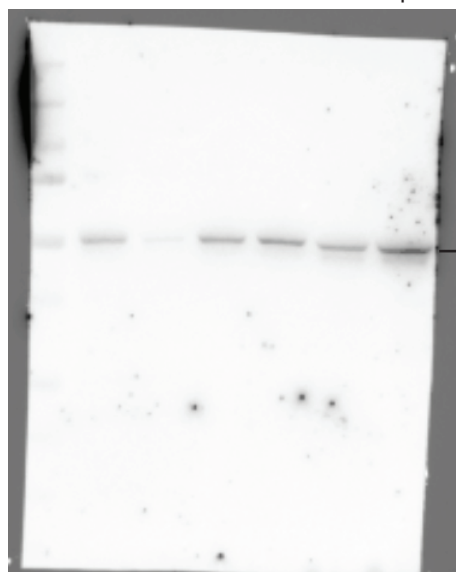

CPK3

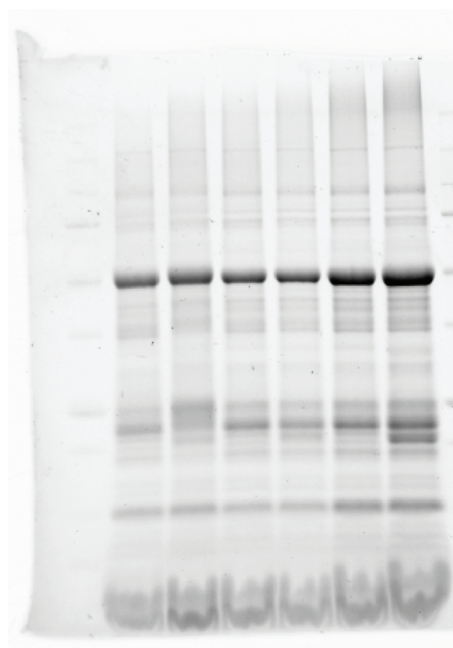

Stain free gel

Supplement: Figure 2—figure supplement 1—source data 3. [file elife-90309-fig2-figsupp1-data3.zip › Figure 2 source data 2/Figure 2 source data 2.pdf]

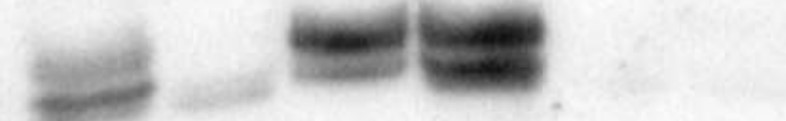

Supplement: Figure 4—figure supplement 1—source data 1. [file elife-90309-fig4-figsupp1-data1.zip › Figure 4 - Figure supplement 1 - Source data 1/Figure 4_Supp1A_blot1.jpg]

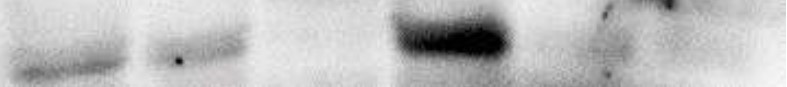

Supplement: Figure 4—figure supplement 1—source data 1. [file elife-90309-fig4-figsupp1-data1.zip › Figure 4 - Figure supplement 1 - Source data 1/Figure 4_Supp1A_blot2.jpg]

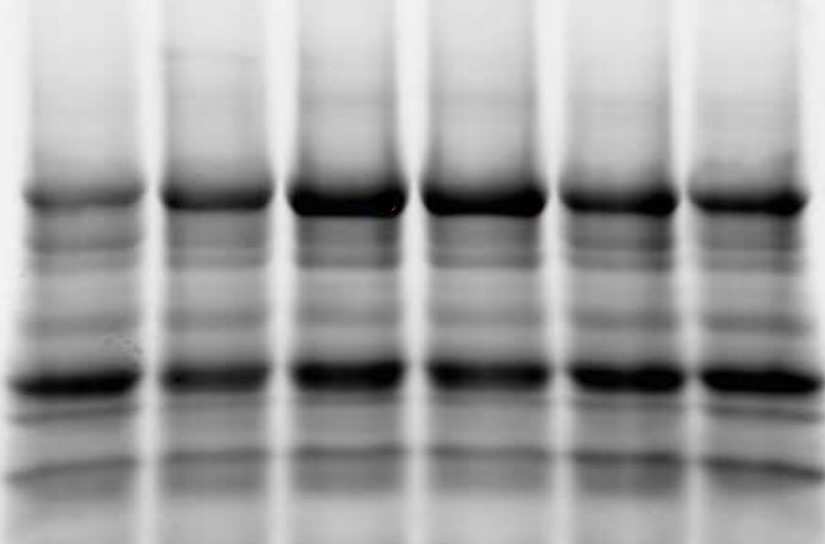

Supplement: Figure 4—figure supplement 1—source data 1. [file elife-90309-fig4-figsupp1-data1.zip › Figure 4 - Figure supplement 1 - Source data 1/Figure 4_Supp1A_coomassie.jpg]

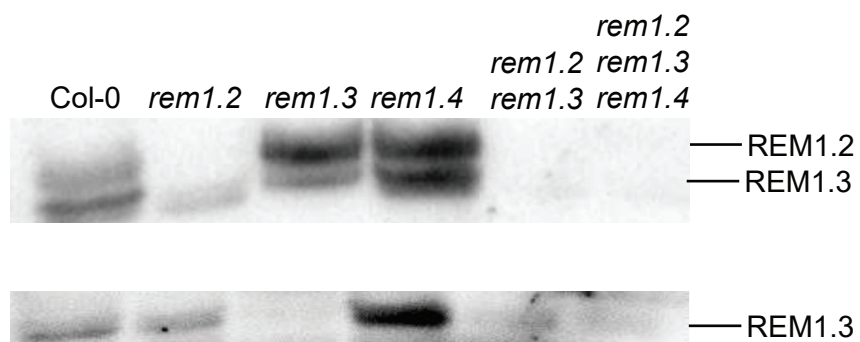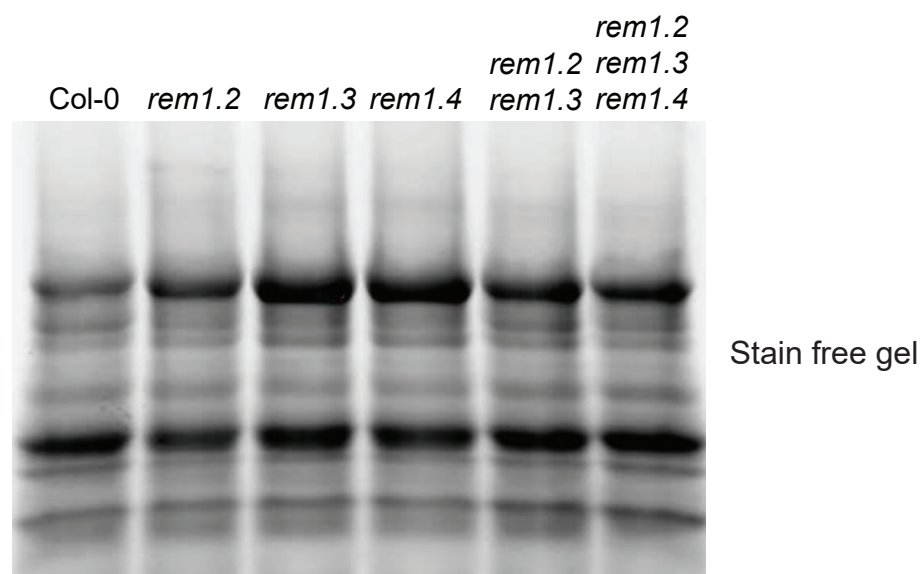

Source data Figure 4 supplemental figure 1 - uncut gel and membrane.

Supplement: Figure 4—figure supplement 1—source data 2. [file elife-90309-fig4-figsupp1-data2.zip › Figure 4 Figure supplement 1 Source data 2/Figure 4 Supplemental Figure 1 Source data 2.pdf]

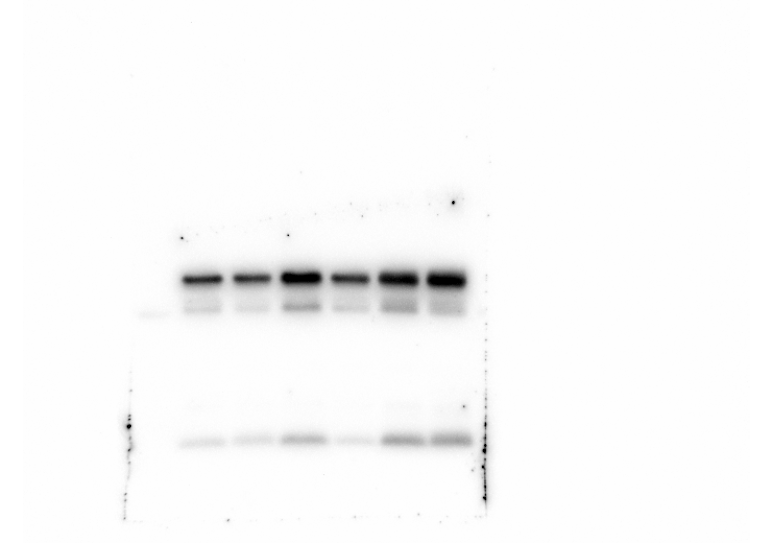

Supplement: Figure 4—figure supplement 3—source data 2. [file elife-90309-fig4-figsupp3-data2.zip › Figure 4 source data 1/membrane4_alphaREM.tif]

| Replicates | #1 |   | #2 |   | #3 |   |
|------------|----|---|----|---|----|---|
| PIAMV      | -  | + | -  | + | -  | + |

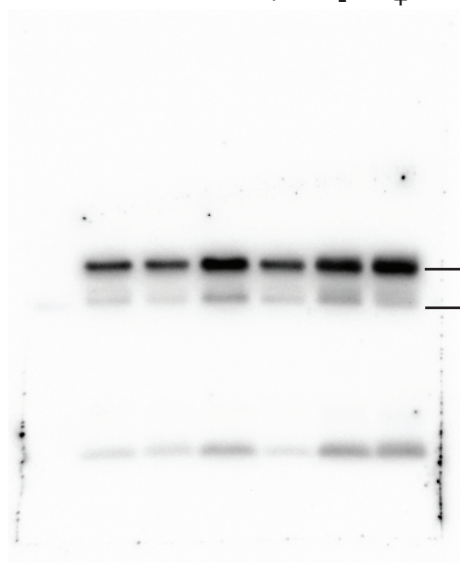

REM1.2  
REM1.3

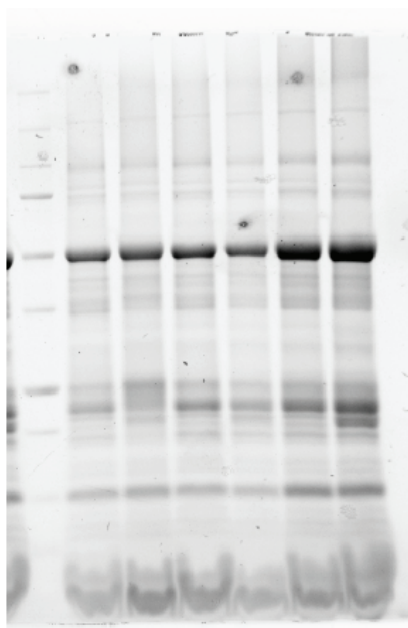

Stain free gel

Supplement: Figure 4—figure supplement 3—source data 3. [file elife-90309-fig4-figsupp3-data3.zip › Figure 4 source data 2/Figure 4 source data 2.pdf]

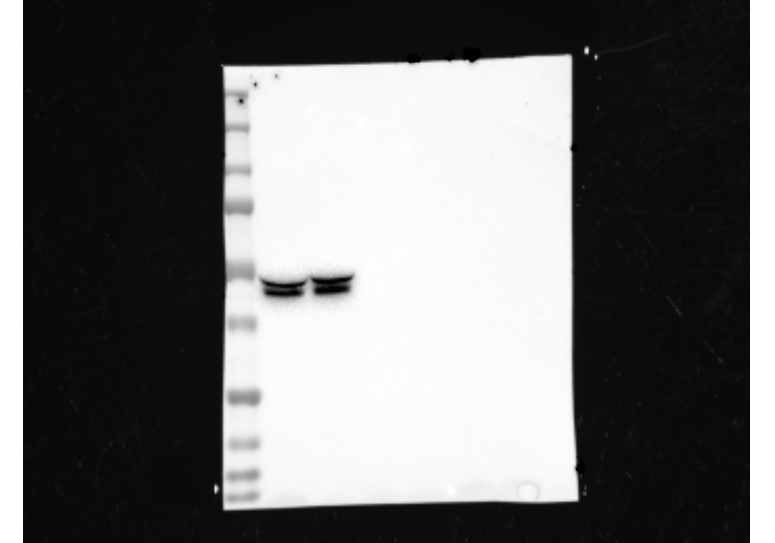

Supplement: Figure 5—figure supplement 1—source data 1. [file elife-90309-fig5-figsupp1-data1.zip › Figure 5 source data 1/CPK3_merged.tif]

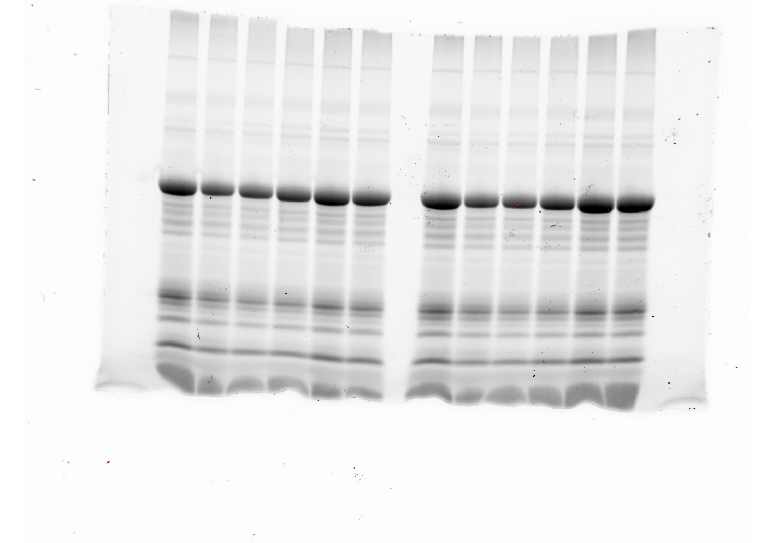

Supplement: Figure 5—figure supplement 1—source data 1. [file elife-90309-fig5-figsupp1-data1.zip › Figure 5 source data 1/gel.tif]

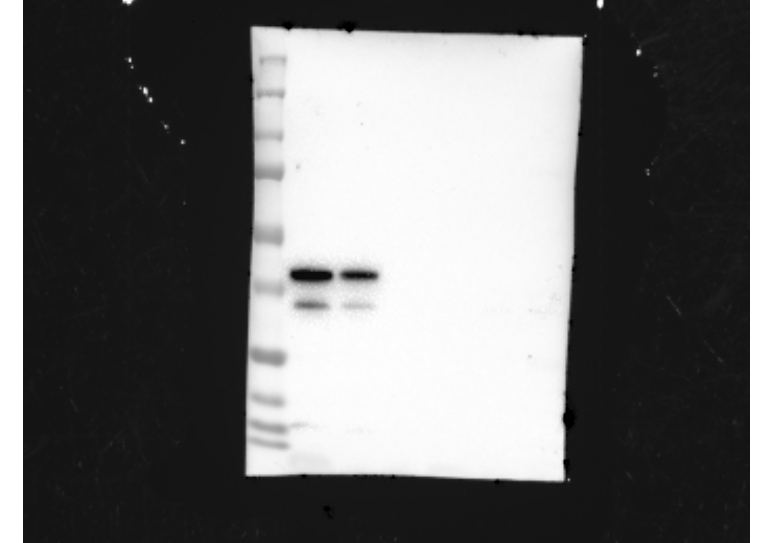

Supplement: Figure 5—figure supplement 1—source data 1. [file elife-90309-fig5-figsupp1-data1.zip › Figure 5 source data 1/REM_merged.tif]
