# Supplementary material for: Interdependence of plasma membrane nanoscale dynamics of a kinase and its cognate substrate underlies Arabidopsis response to viral infection
Source: eLife. 2025 May 2;12:RP90309. doi: 10.7554/eLife.90309 (PMC12048157; doi:10.7554/eLife.90309)
Supplement: Figure 5—figure supplement 1—source data 2. [file elife-90309-fig5-figsupp1-data2.zip › Figure 5 source data 2/Figure 5 source data 2.pdf]

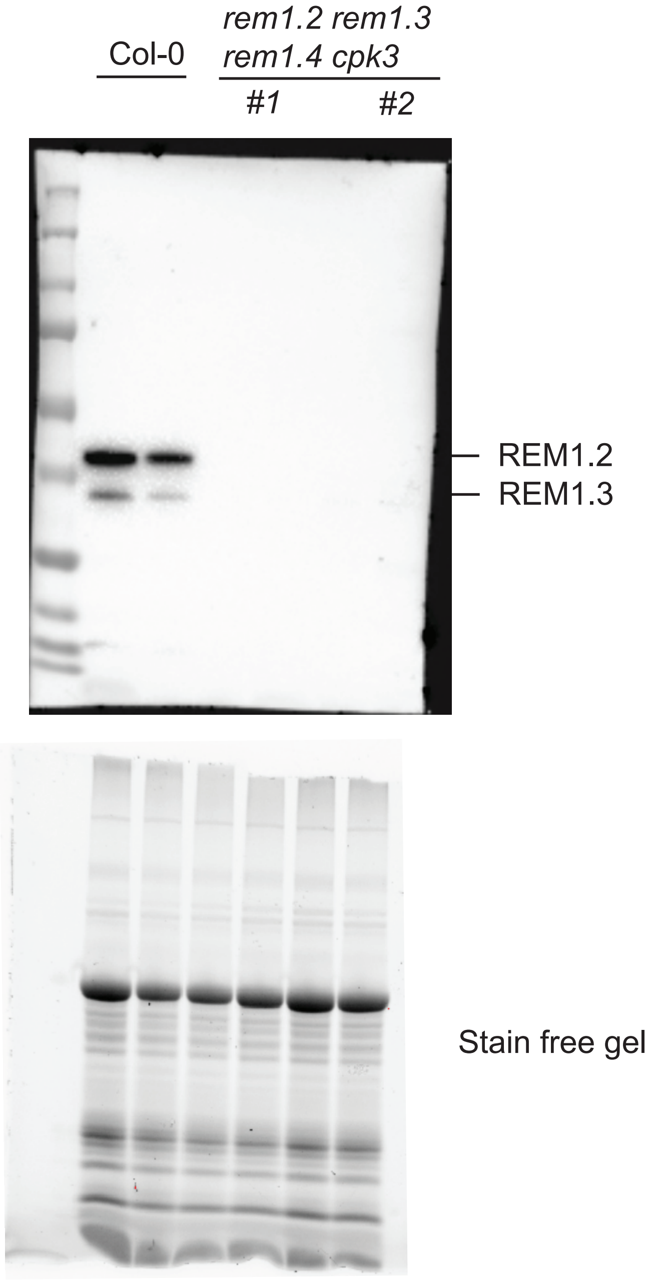
Col-0
rem1.2 rem1.3
rem1.4 cpk3
#1
#2
REM1.2
REM1.3
Stain free gel

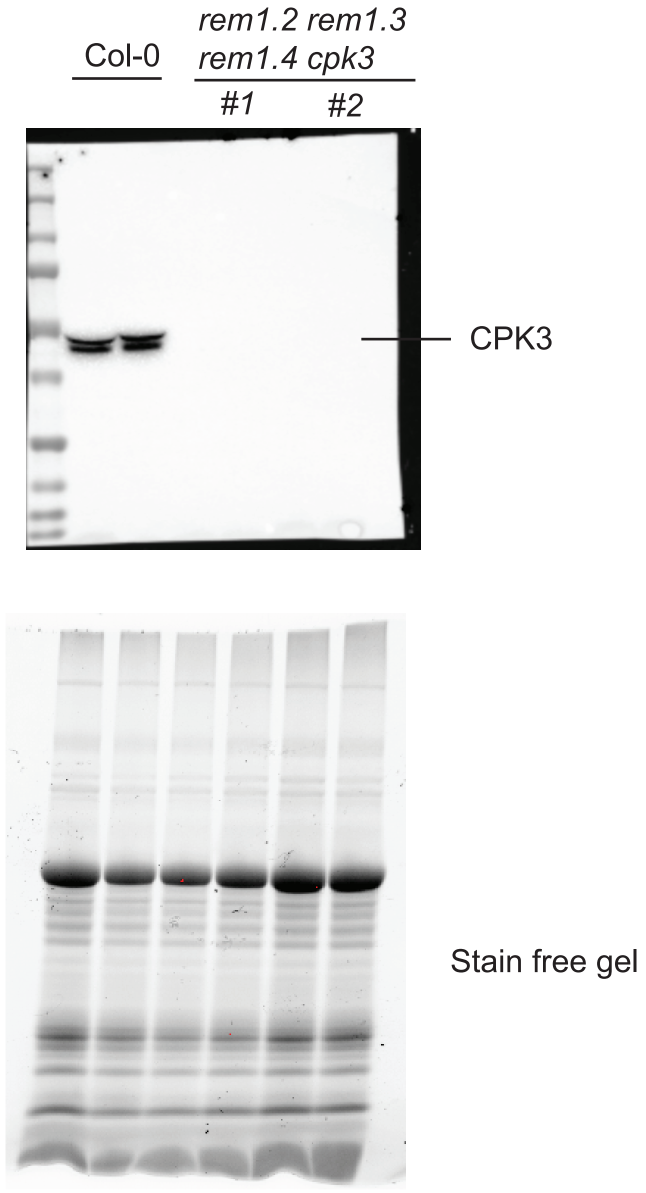
Col-0
rem1.2 rem1.3
rem1.4 cpk3
#1
#2
CPK3
Stain free gel
