# Supplementary material for: Interdependence of plasma membrane nanoscale dynamics of a kinase and its cognate substrate underlies Arabidopsis response to viral infection
Source: eLife. 2025 May 2;12:RP90309. doi: 10.7554/eLife.90309 (PMC12048157; doi:10.7554/eLife.90309)
Supplement: Supplementary file 1. [file elife-90309-supp1.docx]

Supplemental file 1. Primers used in this study

Primers for gateway cloning

| REAGENT or RESOURCE | SOURCE | IDENTIFIER |
| --- | --- | --- |
| GGGG ACA GCT TTC TTG TAC AAA GTG GAA ATG GCG GAG GAA CAG AAG ATA | This study | P2P3_REM1.2_Fw |
| GGGG AC AAC TTT GTA TAA TAA AGT TGG TTA GAA ACA TCC ACA AGT TGC CTT T | This study | P2P3_REM1.2_Rv |
| GGGG ACA AGT TTG TAC AAA AAA GCA GGC TCC ATGGGCCACAGACACAGCAA | This study | P1P2_CPK3_Fw |
| GGGG AC CAC TTT GTA CAA GAA AGC TGG GTC CATTCTGCGTCGGTTTG | This study | P1P2_CPK3_Rv |
| GGGGACAAGTTTGTACAAAAAAGCAGGCTTCGAAGGAGA  TAGAACCATGGGTAATACTTGTGTTG | This study | P5951-CPK1FL-GF |
| GGGGACCACTTTGTACAAGAAAGCTGGGTCTCCACCTCC  GGACTAGAGTTTAAGAGCAATGC | This study | P5952-CPK1FL-GR |
| GGGGACAAGTTTGTACAAAAAAGCAGGCTTCGAAGGAGAT  AGAACCATGGGCAATTCATGTCGT | This study | P3432-CPK6FL-GF |
| GGGGACCACTTTGTACAAGAAAGCTGGGTCTCCACCTCCG  GATCACACATCTCTCATGCTGAT | This study | P3433-CPK6FL-GR |

Primers for Goldengate cloning

| REAGENT or RESOURCE | SOURCE | IDENTIFIER |
| --- | --- | --- |
| ATATATGGTCTCGATTGCCATCACCACCATCCAAGGGTT | This study | CPK3_sgRNA1_Fw |
| TGCCATCACCACCATCCAAGGGTTTTAGAGCTAGAAATAGC | This study | CPK3_sgRNA1_Rv |
| AACATGCGACGGCCTCGGGCATTCAATCTCTTAGTCGACTCTAC | This study | CPK3_sgRNA2_Fw |
| ATTATTGGTCTCGAAACATGCGACGGCCTCGGGCATTCAA | This study | CPK3_sgRNA2_Fw |

Primers for point mutation

| REAGENT or RESOURCE | SOURCE | IDENTIFIER |
| --- | --- | --- |
| AAACAACAGGTCGCTTGCATGTCAATCCCTAC | This study | CPK3_K107M_Fw |
| GTAGGGATTGACATGCAAGCGACCTGTTGTTT | This study | CPK3_K107M_Rv |

Primers for genotyping

| REAGENT or RESOURCE | SOURCE | IDENTIFIER |
| --- | --- | --- |
| AACATGGATAATATCCACGCG | This study | rem1.2_LB |
| TAAGCTCACCTCTATCGAGCG | This study | rem1.2_RB |
| CCACAACTAATTTGTCATGATTCC | This study | rem1.3_LB |
| CACCCGACACTCTGTCAATTC | This study | rem1.3_RB |
| GAAGAGGAACCGAAGAAGGTG | This study | rem1.4_LB |
| TCTCCCATGATCCAATTGAAG | This study | rem1.4_RB |

Primers for RT-qPCR

| REAGENT or RESOURCE | SOURCE | IDENTIFIER |
| --- | --- | --- |
| TTGGTTAGACTCGAGCAAGATAAG | This study | REM1.2_Fw |
| CCCAAGCTCCAACTGAAGAA | This study | REM1.2_Rv |
| GCATCGCTCGATAGAGATGTT | This study | REM1.3_Fw |
| CTCAGCTTTGTTCTCTGCTTTG | This study | REM1.3_Rv |
| GAGATGTGATACTTGCCGACTT | This study | REM1.4_Fw |
| CTGTTCTCAGCCTTTGACTTCT | This study | REM1.4_Rv |
| GGAGAAGCAAGAAGACTCTAAGG | This study | CPK3_Fw |
| GCCAGAACAGCATCTCGATTA | This study | CPK3_Rv |
